# Predictors of clinical response after rTMS treatment of patients suffering from drug-resistant depression

**DOI:** 10.1038/s41398-021-01555-9

**Published:** 2021-11-15

**Authors:** Aurélie Lacroix, Benjamin Calvet, Benjamin Laplace, Marilyne Lannaud, Brigitte Plansont, Sandrine Guignandon, Patrice Balestrat, Murielle Girard

**Affiliations:** 1 Unité de Recherche et d’Innovation, Centre Hospitalier Esquirol, Limoges, France; 2 INSERM, Université de Limoges, CHU Limoges, IRD, U1094 Institut d’Epidémiologie et de Neurologie Tropicale, GEIST, Limoges, France

**Keywords:** Depression, Neuroscience

## Abstract

Repeated transcranial magnetic stimulation (rTMS) is a therapeutic brain-stimulation technique that is particularly used for drug-resistant depressive disorders. European recommendations mention the effectiveness of 30 to 64%. The failure rate of treatment is high and clinical improvement is visible only after a certain period of time. It would thus be useful to have indicators that could anticipate the success of treatment and more effectively guide therapeutic choices. We aimed to find predictive indicators of clinical improvement at 1 month after the start of rTMS treatment among the data collected during the care of patients with drug-resistant depression included in the Neuromodulation Unit of the Esquirol Hospital in Limoges since 2007. In total, 290 patients with a pharmaco-resistant depressive episode, according to the Hamilton Depression Rating Scale (HDRS) (score ≥8), before treatment who underwent a complete course of rTMS treatment and did not object to the use of their collected data were included. The clinical response in routine practice, corresponding to a decrease in the HDRS score of at least 50% from inclusion, was determined and complemented by interquartile analysis. A combination of factors predictive of clinical response during care, such as a short duration of the current depressive episode associated with a higher HDRS agitation item value (or a lower perceived sleepiness value) and a higher number of previous rTMS treatments, were identified as being useful in predicting the efficacy of rTMS treatment in routine clinical practice, thus facilitating the therapeutic choice for patients with drug-resistant depression.

## Introduction

Repeated transcranial magnetic stimulation (rTMS), based on the principle of electromagnetic induction, is a brain-stimulation technique developed in the 1980s for the purpose of electrophysiological exploration. Technical progress has allowed this technique to evolve towards stimulation for therapeutic purposes, particularly in psychiatry. The effectiveness of stimulation of the right prefrontal cortex at low frequency or the left prefrontal cortex at high frequency for the treatment of depressive disorders and the clinical tolerance were established in the early 2000s. As a result, rTMS has been recommended for the management of drug-resistant episodes (Food and Drug Administration; 2008 and Canadian Network for Mood and Anxiety Treatments; 2009) [1, 2]. European recommendations mention an efficacy of 30 to 64% (Grade A recommendation) [3, 4] but are unsure about its place in the therapeutic strategy for depression.

Indeed, in clinical practice, the first-line treatment for depressive disorder (excluding bipolar disorder) is still the use of antidepressants (such as selective serotonin reuptake inhibitors (SSRIs) or serotonin and norepinephrine reuptake inhibitors (SNRIs)), alone or in combination with psychotherapy. Their effectiveness is between 40 and 50%, depending on the criteria used. However, a poor response to antidepressant therapies is one of the risk factors for developing chronic depression. The management of such resistant depressive episodes is, therefore, an important issue. The therapeutic strategy in situations of nonresponse to the first line of treatment is still unclear, as is the strategy after failure of the second line of treatment.

rTMS may represent an alternative by providing a clinical response for people in depressive episodes confronted with the failure of pharmacological antidepressant treatment. Given the time of action of antidepressants (clinical response after 3 weeks), as well as the duration of rTMS treatment (clinical effect at 2 or even 3 weeks), it is necessary to identify factors that can predict the success of the treatment in advance and more effectively guide therapeutic choices. Such factors are mentioned in the literature and can be divided into two main categories: “patient-related” factors and those “related to the characteristics of the depressive episode itself “.

Among patient-related factors, data in the literature on the efficacy of rTMS according to the age or gender of the patients are discordant. Indeed, a better response to rTMS treatment has been observed in young individuals (<45 years) [5, 6], whereas other studies have found no correlation with age (< or >65 years) [7]. In addition, female gender may be a factor in the positive response to treatment, but has been shown in only one isolated study [8].

Among factors related to the characteristics of the depressive episode itself, the efficacy of rTMS may be associated with the duration of the depressive episode, severity of the depression (based on Hamilton Depression Rating Scale (HDRS) or Beck Depression Inventory (BDI) scores), diagnosis (established according to International Classification of Diseases-10 (ICD-10)), therapeutic resistance, number of rTMS cures, or history of electroconvulsive therapy (ECT). Indeed, a short duration of the current depressive episode (<5 months) appears to favor a clinical response after treatment with rTMS, with a 50% decrease in the HDRS score as the gold standard for defining this clinical response, complemented by a quartile analysis of the population using the same scale. Within this scale, the depressive sub-core [9–11], sleep [12], and anxiety [13] are often found to be improved by the various treatments (imipramine, fluoxetine, duloxetine, ECT, rTMS, etc.). In terms of diagnosis, there is clearly no difference in efficacy between the treatment of uni and bipolar depression, but this is debated [14]. Finally, a low number of previous rTMS treatments [15] or good response to ECTs [16] may be predictive of a good response of patients to rTMS.

We conducted a retrospective naturalistic study in the Neurostimulation Department of the Esquirol Hospital in Limoges on drug-resistant depressive patients from an active file of more than 600 patients referred for rTMS for various pathologies since 2007. We analyzed the scores for the clinical global response (CGI), BDI, HDRS, and perceived deficits questionnaire within this population, with a clinical response defined as a 50% decrease in the initial HDRS score (D0) by either D15 and/or M1. The evolution of the clinical response from D0 to M1 was also studied. Patients with resistant depression were finally examined to identify factors predictive of clinical response at 1 month (M1) after the beginning of rTMS treatment.

**Fig. 1:**
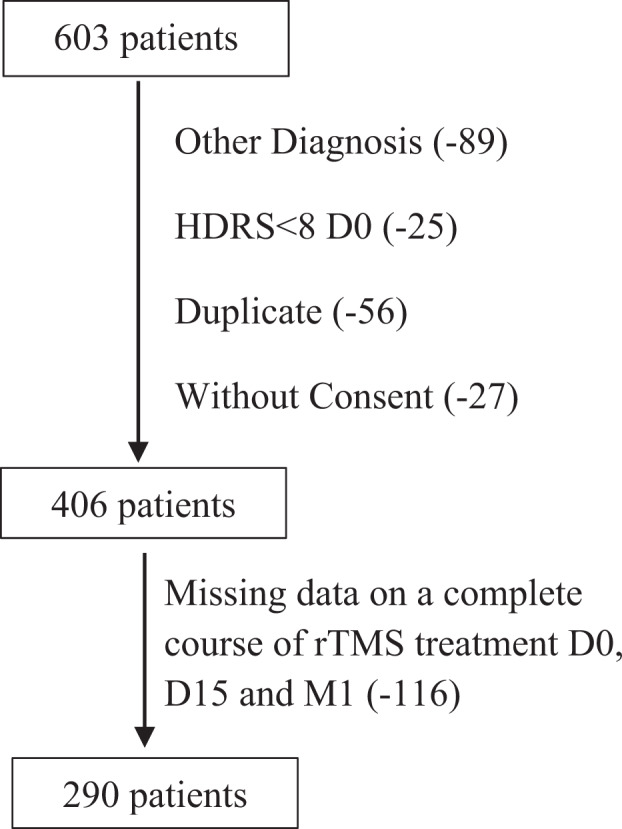
Flow chart of the study.

## Methods

### Population (Fig. 1)

Included in the study were:

- patients over 18 years of age with a diagnosis of uni- or bipolar depression, according to the DSM-IV-TR and DSM-5 criteria since 2016: specifically, patients with the bipolar affective disorder (F31 ICD-10), depressive episodes (F32 ICD-10), recurrent depressive disorder (F33 ICD-10), or persistent [affective] mood disorders (F34 ICD-10).
- patients with depressive episode characterized by an HDRS score ≥8 at D0 and drug-resistant (at least minor and/or major), i.e., the failure of at least two drug treatments prior to their treatment with rTMS. More specifically, a minimum HDRS score of 16 at D0 is generally considered to be the reference to indicate moderate depression [17]. Thus, we distinguished between patients with so-called mild depression, with an HDRS score between 8 and 15, and those with major depression, with an HDRS score ≥16.
- patients undergoing a complete course of rTMS treatment (low frequency, right dorsolateral prefrontal cortex stimulation) comprised of five sessions per week for 3 weeks associated with three follow-ups on D0, D15, and M1.

The only contraindications were those classically associated with rTMS treatment:

- presence of a pacemaker

- a cardiac or cerebral stimulator

- metal splinters in the head

- brain injury

- active comitiality

- pregnancy

- intracranial hypertension.

Participation in another biomedical research study was not allowed.

The clinical evaluations and corresponding data have been collected since 2007 in dedicated files. Non-opposition to the use of the participants’ research data was obtained retrospectively for those included before 2018 and prospectively since. All patients provided informed written consent and the study received legal authorizations from the Committee for the Protection of Persons and the French Agency for the Security of Health Products.

### rTMS treatment

The treatment included five daily sessions per week for 3 weeks. A daily session consisted of 20 min of stimulation at a low frequency of 1 Hz (Magstim Super Rapid^2^, Inomed, Emmendingen, Germany)), with 120 trains of 10 s of stimulation (60 pulses) and an inter-train interval of 1 s. This corresponded to 1200 stimulations per session at 100% of the resting motor threshold (RMT). The cerebral area stimulated was the right dorsolateral prefrontal cortex. A neuronavigation process (Visor 3D, ANT, Enschede, Netherland) allowed real-time monitoring of the specific position of stimulation based on medical imaging obtained by magnetic resonance imaging (MRI) (Asalab, ANT, Enschede, Netherland).

### Psychometric evaluations

The CGI [18] is a global evaluation that uses three independent item scales. It is easy to use and generalizable to all pathologies or comorbidities. The scales consist of the severity of illness (0 to 7), the global clinical response after treatment (0 to 7), and a composite score on a four-point scale that addresses treatment efficiency and adverse effects.

The score of item 1 of the CGI on D0 and the scores of items 1 and 2 at D15 and M1, taken separately, were analyzed.

The BDI is a 21-item, self-reported rating inventory that measures the intensity of depression [19]. Each item is comprised of four sentences corresponding to four increasing degrees of intensity relative to a symptom and is scored from 0 to 3. The highest score obtained is that selected for the same set. The global score is determined by summing the scores of the 21 items. A score <10 indicates the absence of depression, from 10 to 18 mild depression, from 19 to 29 moderate depression, and >30 severe depression.

The overall BDI score, as well as the score for items L (Fatigue) and M (Appetite) at D0, D15, and M1 were analyzed.

The HDRS [20, 21] is a 17-item instrument that was designed to measure the frequency and intensity of depressive symptoms in individuals with major depressive disorder completed by the clinician. Ratings are made using a Likert scale of either 0 to 4 or 0 to 2 for each item, yielding total scores from zero to 52. HDRS scores are classified as normal (0 to 7), mild depression (8 to 16), mild to moderate depression (17 to 23), and moderate to severe depression (>24).

The overall HDRS global score, sleep (items 4 + 5 + 6) and anxiety (items 10 + 11) sub-scores, and the depressive core (HDRS_6_:1 + 2 + 7 + 8 + 10 + 13), in addition to the score for items 8 (slowdown), 9 (agitation) 13, (general symptoms), 15 (hypochondria), and 16 (weight loss) at D0, D15, and M1 were analyzed.

The questionnaire on perceived deficits [22] includes five items that can be scored from 0 to 4 for each (0 corresponds to not once and 4 to very often). This questionnaire concerns memory, attention, or concentration problems that some people may have. These situations are assessed based on a maximum score of 20 in the last 7 days.

### Clinical response

The gold standard definition for the clinical response, consistent with the data in the literature, consisted of a calculated clinical response of at least a 50% reduction in the HDRS score between the assessment at inclusion and those on D15 and/or M1.

As this was a retrospective naturalistic study on the data collected, it is possible that the parameters used to select and qualify patients and their evolution could be different from those corresponding to international criteria, such as the gold standard using a decrease in the HDRS score of at least 50% relative to inclusion to judge the effectiveness of treatments. Thus, the clinical response calculated according to the gold standard was complemented by a calculation of the population clinical response using the same quartile HDRS score ratio (D15 and M1 assessments versus inclusion) all encoded in “clinical response” or “no clinical response”. Patients presenting a net benefit of rTMS treatment (located in the lower quartile, i.e., the first 25% of the population) were therefore notably analyzed. Thus, the first defined groups were observed completed by an analysis in the lowest quartile all with a binary clinical response based on a decrease of at least 50% of the HDRS score.

### Statistical analysis

Quantitative variables are presented as the mean and standard deviation. Qualitative variables are presented as percentages and counts. Intergroup comparisons for the quantitative variables were made using Mann–Whitney nonparametric tests for a given time point. The Chi-square test was used to compare groups for the qualitative variables. Analyses over time were performed to describe the clinical changes with a matched nonparametric Wilcoxon test for analyses between two-time points. Finally, a binary stepwise logistic regression model was generated to identify the predictive factor(s) (variables collected at D0 as a predictive factor of the clinical response at M1). Variables that differed between clinical response groups with *p* < 0.2 were introduced into the regression model. Results with p values <0.05 were considered significant. Analyses were performed using SPSS Statistics 27.0 software (IBM).

**Table 1:** Descriptive data at inclusion.

Age (years) (mean ± SD)		55.1 ± 12.6
Sex (*n* (%))	Men	97 (33.3)
	Women	194 (66.7)
Attached (*n* (%))	Yes	129 (44.3)
	No	159 (54.6)
Children (*n* (%))	Yes	188 (64.6)
	No	99 (34.0)
Work (*n* (%))	Yes	120 (41.2)
	No	161 (55.3)
Diagnosis (*n* (%))	F31	39 (13.4)
	F32	86 (29.6)
	F33	155 (53.3)
	F34	11 (3.8)
ECT (*n* (%))	Yes	27 (9.3)
	No	264 (90.7)
Previous treatments (mean ± SD)	Antidepressant number	3.0 ± 2.1
	Total number	4.7 ± 2.5
Current depressive episode (mean ± SD)	Duration in months	7.2 ± 11.2
BDI total score (mean ± SD)		19.6 ± 6.4
HDRS total score (mean ± SD)		17.4 ± 4.4
CGI1 (mean ± SD)		5.3 ± 0.6
Perceived deficits questionnaire (mean ± SD)		9.9 ± 5.3
Sleeping	Yes	21 (7.2)
	No	270 (92.8)

## Results

### Population at inclusion (Table 1)

In total, 291 patients (194 females and 97 males) were included. The characteristics of the studied population at inclusion are presented in Table 1. The mean age was 55.1 ± 12.6 years (range 24 to 88 years). Nearly half of the patients (54.6%, *n* = 159) were married or in a long-term relationship but without children (64.6%, *n* = 188), and 55.3% had a professional activity (*n* = 161). Most of the diagnoses corresponded to a recurrent depressive disorder (53.3%, *n* = 155), followed by isolated depressive episodes (29.6%, *n* = 86), bipolar affective disorder (13.4%, *n* = 39), and persistent [affective] mood disorders (3.8%, *n* = 11). Most patients (90.7%, *n* = 264) had not received ECT prior to rTMS treatment but had undergone several courses of antidepressant treatment (*n* = 3.0 ± 2.1) with a wide variety of drugs (*n* = 4.7 ± 2.5). Patients presented with a current depressive episode of 7.2 ± 11.2 months’ duration characterized by moderate depression according to both the HDRS and BDI criteria (17.4 ± 4.4 and 19.6 ± 6.4, respectively). The score for the first item of the CGI was high (5.3 ± 0.6), as was the response to the perceived deficits questionnaire (9.9 ± 5.3). The frequency of adverse effects of rTMS treatment, such as sleepiness, was low (7.2%, *n* = 21).

**Table 2:** Evolution of scores from D0 to M1.

HDRS total score (mean ± SD)	D0		17.4 ± 4.4	
	D15		9.6 ± 4.7	
	M1		9.6 ± 5.7	
Clinical response (*n* (%))	Gold standard: decreasing 50% HDRS			
	D15		Yes	127 (43.6)
			No	164 (56.4)
	M1		Yes	128 (44)
			No	163 (56)
	Interquartiles: first 25% vs last 25%			
	D15		Yes	73 (25.1)
			No	74 (25.4)
	M1		Yes	76 (26.1)
			No	74 (25.4)
BDI total score (mean ± SD)	D0		19.6 ± 6.4	
	D15		10.5 ± 6.6	
	M1		9.9 ± 6.7	
CGI	1	D0	5.3 ± 0.6	
		D15	4.7 ± 1.0	
		M1	4.3 ± 1.0	
	2	D15	2.8 ± 0.9	
		M1	3.4 ± 1.0	
Perceived deficits questionnaire	D0		9.9 ± 5.3	
	D15		7.3 ± 4.3	
	M1		7.7 ± 4.8

**Fig. 2:**
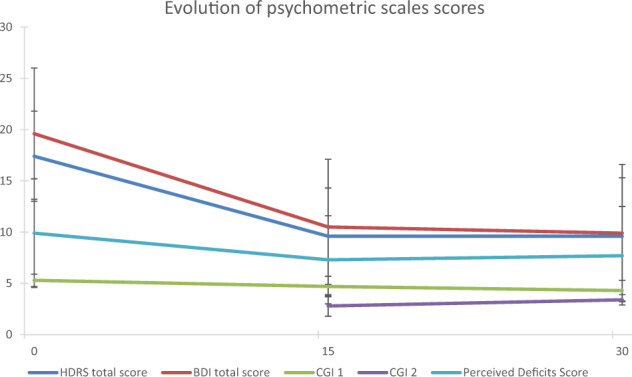
Evolution of psychometric scales scores from D0 to M1.

### Evolution of scores from D0 to M1 (Table 2)

#### Evolution of psychometric scales scores (Fig. 2)

The total HDRS score decreased significantly from D0 to D15 (*p* < 0.001) before stabilizing by M1 (*p* = 0.562), along with the score from the perceived deficits questionnaire (D0 to D15, *p* = 0.005, D15 to M1 *p* = 0.716). The total score of the BDI, as well as that of the first item of the CGI relative to illness severity, decreased significantly from D0 to D15 (*p* < 0.001 for both) and from D15 to M1 (*p* = 0.003, *p* < 0.001, respectively). The second item of the CGI relative to the global clinical response after treatment showed a significant increase between D15 and M1 (*p* < 0.001).

**Fig. 3:**
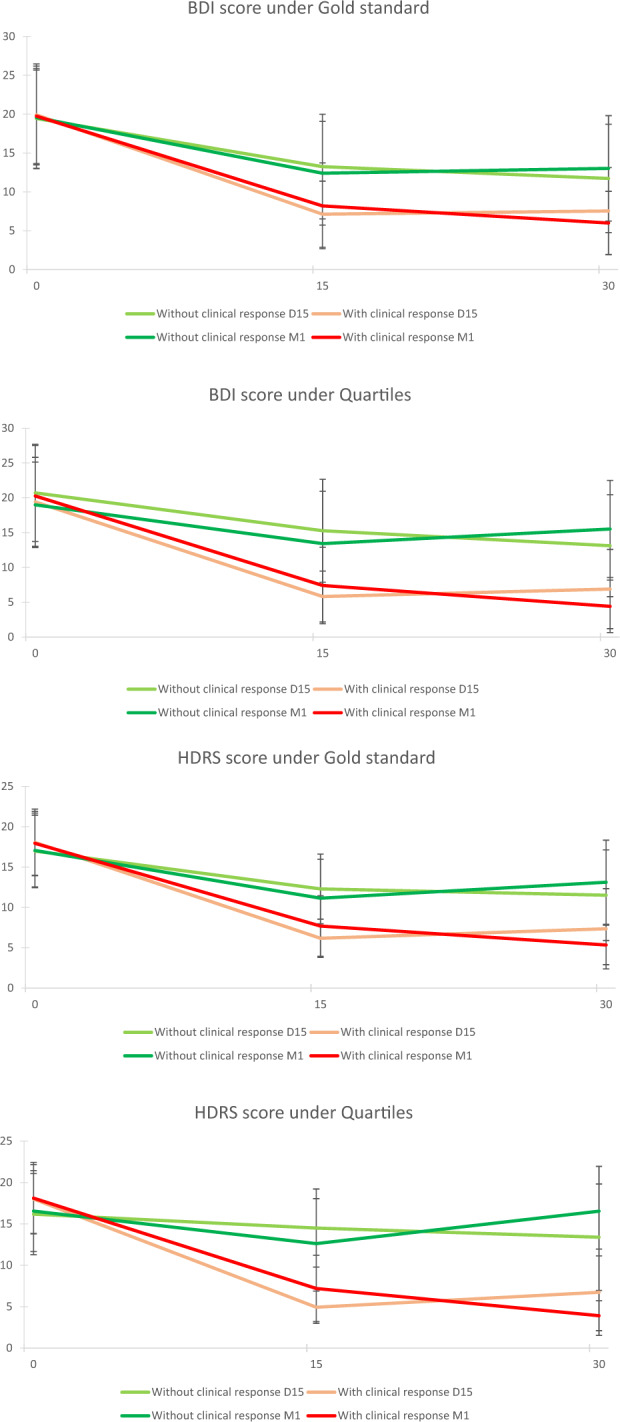
Clinical response based on the evolution of the HDRS and BDI psychometric scales scores under gold standard and quartiles analysis.

#### Clinical response based on the evolution of the psychometric scales (HDRS and BDI) scores (Fig. 3)

The profiles established from the calculation of the clinical response at D15 can be distinguished from those established from the calculation of the clinical response at M1 based on their evolution from D15 to M1.

Indeed, the calculation of the clinical response at D15 and M1 showed a significant decrease (*p* < 0.001) in the total scores of the BDI and HDRS scales from D0 to D15, regardless of the group considered (with or without a clinical response), whereas from D15 to M1, there was a significant decrease in the HDRS scores in the group with clinical response and a significant increase in the HDRS score and a nonsignificant tendency towards an increase in the BDI score for the group without clinical response. The analysis previously performed is reversed between the groups identified from the calculation of the clinical response at D15.

The profiles of the observed evolution were identical regardless of how the clinical response was calculated.

There was no difference in the BDI score between the groups with and without a clinical response at D0. The same was true for the HDRS score but only for the clinical response calculated according to the gold standard at M1. All observable differences for the BDI and HDRS were significant at D15 and M1. Thus, based on the clinical response calculated at M1, from which two groups could be identified (one with and one without a clinical response) and despite the significant previously described variation in scores, there was a greater difference between the scores at M1 than at D15. However, the difference was greater at D15 than M1 if the calculated clinical response at D15 was used.

The profiles described above were even more pronounced if the quartile method to judge the clinical response was used.

### Clinical response

Clinical response was observed on D15 for 43.6% of the population (*n* = 127) considered as responders. A comparable clinical response (*p* = 0.913) was observed at M1 for 44% (*n* = 128).

Four categories were statistically distinguishable (*p* < 0.001) for clinical responses observed at D15 and M1. The absence of a clinical response over time was observed for 41.9% of the population (*n* = 122). Clinical response at D15 only was observed for 14.1% of the population (*n* = 41) and comparable to that observed at M1 for 14.4% of the population which had not improved by D15 (*n* = 42). A persistent clinical response (D15 and M1) over time was observed for 29.6% of the population (*n* = 86). An interquartile analysis led to identical conclusions, with similar proportions.

**Table 3:** Predictive factors (stepwise binary logistic regression) at M1 under gold standard (a) and quartiles (b) analysis.

a						
	β	ES	Wald	ddl	*p*	Exp(B)
Duration current depressive episode	−0.084	0.034	6.143	1	0.013	0.920
HJ0 item 9	0.567	0.276	4.206	1	0.040	1.762
Number of previous cures	0.718	0.381	3.547	1	0.060	2.050
global % = 69.2, χ² = 18.008, *R*^2^ Nagelkerke = 0.143, ddl = 3, *p* < 0.001.						

b						
	β	ES	Wald	ddl	*p*	Exp(B)
Number of previous cures	0.826	0.350	5.562	1	0.018	2.283
HJ0 item 9	0.521	0.272	3.664	1	0.056	1.684

global % = 73.3, χ² = 9.264, *R*^2^ Nagelkerke = 0.081, ddl = 2, *p* = 0.010.

### Predictive factors at M1 (Table 3)

Among the variables (age, number of antidepressant treatments, number of drugs, BDI D0 (total, item L and M), HDRS D0 (total, sleep and anxiety sub-cores, depressive core, items 8-9-13-15-16), CGI1 D0, the number of previous courses of rTMS, duration of the current depressive episode, perceived deficits at D0, sleepiness, diagnosis, professional activity, married or in a long-term relationship, children, ECT prior to rTMS treatment, and sex) those with *p* < 0.2 based on the clinical response criteria at M1 were introduced into a regression model. They included the duration of the current depressive episode (group without a clinical response: 8.88 ± 13.59 months/group with a clinical response: 4.77 ± 5.29 months, *p* = 0.002), the score for item 9 (“agitation”) of the HDRS at D0 (group without a clinical response: 0.30 ± 0.66/group with a clinical response: 0.45 ± 0.75, *p* = 0.052), the number of previous courses of rTMS (group without a clinical response: 1.16 ± 0.54/group with a clinical response: 1.24 ± 0.54, *p* = 0.065), sleepiness (group without a clinical response: 0.10 ± 0.31/group with a clinical response: 0.03 ± 0.17, *p* = 0.017), and diagnosis (*p* = 0.180). After the introduction of these variables into a stepwise binary logistic regression, the duration of the current depressive episode, alone or in combination with the HDRS item 9 score, or the two in combination with the number of previous courses of rTMS, were predictive of the patient’s classic clinical response at M1.

However, the duration of the current depressive episode combined with the HDRS item 9 score and the number of previous courses of rTMS explained 14.3% of the variance of the clinical response at M1. The regression coefficient associated with the duration of the current depressive episode was significantly negative (ß = −0.84, *p* = 0.013), whereas the regression coefficients associated with the HDRS item 9 score (ß = 0.567, *p* = 0.040) and the number of previous courses of rTMS treatment (ß = 0.718, *p* = 0.060) were positive. Thus, a short duration of the current depressive episode combined with a strong “agitation” parameter and a large number of previous rTMS treatments (*p* < 0.001) was the most discriminating combination of factors predictive of the patient’s standard clinical response at M1 among those observed (Table 3a).

We next used the quartile method and examined the first 25% of the population, corresponding to the patients who improved the most to focus on those patients who improve the most and to characterize them more finely concerning the factors that are predictive of their clinical response. Using the same regression analysis, we verified that the three variables previously found before their introduction into the regression had a *p* < 0.2. Thus, the duration of the current depressive episode (group without a clinical response: 8.99 ± 17.37 months/group with a clinical response: 5.04 ± 6.01 months, *p* = 0.085), the HDRS item 9 score (group without a clinical response: 0.22 ± 0. 56/group with a clinical response: 0.50 ± 0.76; *p* = 0.017), and the number of previous courses of rTMS (group without a clinical response: 1.24 ± 0.72/group with a clinical response: 1.30 ± 0.63, *p* = 0.054) were analyzed for their predictive value. After performing a stepwise binary logistic regression, the number of previous courses of rTMS, alone or in combination with the HDRS item 9 score, was the most convincing predictor combination of the patients’ strong clinical response at M1. The number of previous courses of rTMS combined with the HDRS item 9 score explains 8.1% of the variance of the clinical response at M1. The regression coefficients associated with the number of previous courses of rTMS (ß = 0.826, *p* = 0.018) and the HDRS item 9 score (ß = 0.521, *p* = 0.056) were positive. Thus, a large number of previous rTMS cures associated with a parameter of high “agitation” (*p* = 0.010) was the most discriminating predictor of a strong clinical response at M1 among those observed (Table 3b).

We also observed a negative correlation of the “sleepiness” parameter with the clinical response at M1, regardless of the method used to calculate the clinical response (*p* = 0.016).

## Discussion

This retrospective and naturalistic study demonstrates the relative efficacy of rTMS in terms of clinical response 1 month after the beginning of treatment of 291 patients suffering from drug-resistant depression treated at the Neuromodulation Department of the CH Esquirol Limoges. In addition, it allowed us to identify a combination of factors predictive of this clinical response in the care setting.

We defined resistant depression, as in the literature, by the failure of more than two previous courses of treatment [23], equal to five in our study, all lines combined, including three with antidepressants. In addition, a recommendation for the indication of rTMS for the treatment of depressive episodes characterized by the failure of at least one antidepressant drug treatment was already published in 2014 [3].

We defined a clinical response as a 50% reduction in the HDRS score. We chose this definition because it constitutes a gold standard in the literature, as numerous publications have used it [7], including originally in a study by Brakemeier et al. [24].

The clinical response based on the criterion linked to a 50% reduction in the HDRS score was confirmed by the evolution of the scores for the various scales analyzed in this study from D0 to M1. Indeed, we observed a significant decrease in the HDRS score from D0 to D15, showing the effectiveness of rTMS on the intensity of depression, which was stable up to M1 for some patients, showing maintenance of the effectiveness of this approach over time. This was corroborated by an identical evolution of the deficits perceived by the patient. The effectiveness of rTMS was also corroborated by the BDI and CGI item 1 scores, which measure the intensity of the depression and the severity of the disease according to the patient’s own statements, respectively. According to the patient’s own statements, the intensity of depression, as well as the severity of the disease, decreased significantly from D0 to D15 and also from D15 to M1. The association of such a decrease with the effectiveness of the rTMS method itself was further established by CGI item 2, which showed a significant increase that corresponded to the effectiveness of the treatment. Hetero-questionnaires are often preferred to self-questionnaires, although the latter provide another type of information concerning the patient’s own feelings. Thus, although we used the HDRS as the basis for the calculation of the clinical response because it is highly stable and very often referenced in publications, we also assessed the BDI as a parameter in its own right, without using it for the calculation of the clinical response. Indeed, although other publications have used a 50% reduction in the overall score of the BDI, they are still few in number and highly controversial [25].

More generally, the evolution of the clinical response showed M1 to be better than D15 for the calculation of the clinical response. Indeed, the values at M1 that defined the groups with or without a clinical response based on the HDRS were more discriminant, as were those of the BDI, which is used to measure the intensity of depression, as already mentioned. The discrimination between the two groups was even clearer using the quartile method.

Here, we found that more than 40% of the population had improved by D15 or M1 using this criterion. This criterion for the clinical response, therefore, appears to be stable over time based on these figures but does not demonstrate the evolution of this clinical response, i.e., whether the same patients who showed improvement on D15 also did so at M1.

Comparison of the data of the clinical response at D15 and M1 distinguished four groups (*p* < 0.001). Finally, only 30% of the population showed a clinical response over time from D0 to M1, complemented by the fact that 14% of the population showed only an improvement on D15 or only at M1. In conclusion, 40% of the population did not improve at all over time, but nearly 60% of the population showed a clinical response for at least one time point over this period, demonstrating the efficacy of rTMS on drug-resistant depression [4].

It was thus possible to define the factors predictive of this clinical response based on the clinical response at M1 by regression and correlations. After carrying out the regression we found the combination of a short duration of the depressive episode, a higher value of the item 9 score of the HDRS at inclusion, which relates to agitation, and a higher number of previous courses of rTMS to be the best combination to predict the effectiveness of rTMS. This combination of factors has not been formerly demonstrated in the literature and may be of great utility, as each of these factors has already been individually shown [26] to be a predictor of the effectiveness of rTMS. Indeed, several studies [24, 27–29] concluded that a short duration of the current depressive episode favors a good clinical response to treatment with rTMS, with an established duration of <5 months. We found the duration of the current depressive episode to be 4.77 months in the improving group, confirming the use of this factor as a predictor, as well as its use in this combination of predictive factors.

Concerning the HDRS item 9 score, stronger agitation could indicate greater vigilance on the part of the patient, greater attention to his/her condition, and his/her being more alert, as the score was between 0 and 1 in our study, corresponding to muscle contractions synonymous with psychomotor agitation of the subject. Fitzgerald et al. [30] indeed demonstrated that the level of psychomotor agitation positively correlates with the response to rTMS treatment. This can be compared to the significant negative correlation, also made at M1, concerning a decrease in the observation of sleepiness during the rTMS session.

Concerning the higher number of previous courses of rTMS, Kelly et al. [15] concluded that patients who responded well to the first course of rTMS during a depressive episode were also good responders for later episodes. It is also known that maintenance sessions are critical for stabilizing the clinical response of the depressive episode [31]. Thus, repeated courses of rTMS appear to have a positive effect on the patient’s clinical response, reinforcing our analysis.

We focused on the 25% of patients who improved the most in the population based on the quartile method to complete our analysis of the relevance of this combination of predictive factors. This approach identified a higher number of previous courses of treatment and increased psychomotor agitation to be the most robust predictors of a strong clinical response at M1. At the same time, the significant correlation with a decrease in sleepiness may still be related and compared with the score for item 9 of the HDRS. The duration of the current depressive episode appears to be a less robust criterion, but the inclusion of a larger number of patients could allow this criterion to be added to the other two, as in the first gold-standard analysis. Indeed, the duration of the current depressive episode evolved in the same direction based on the quartile analysis, with a comparable difference between the groups with or without clinical response, without, however, being significant due to a too large disparity in values in such a small population.

The contribution of these factors to the prediction are relatively small but highly significant, giving them important value for further prospective studies with larger sample size.

We were able to define a combination of factors that predict the clinical response in the care setting that could be extremely useful in predicting the efficacy of rTMS treatment in routine clinical practice, leading to clearer therapeutic choices for patients with drug-resistant depression in the neuromodulation service.
